# Reoxygenation of Asphyxiated Newborn Piglets: Administration of 100% Oxygen Causes Significantly Higher Apoptosis in Cortical Neurons, as Compared to 21%

**DOI:** 10.1155/2014/476349

**Published:** 2014-03-25

**Authors:** G. Faa, V. Fanos, D. Fanni, C. Gerosa, A. Faa, M. Fraschini, M. E. Pais, E. Di Felice, A. Papalois, M. Varsami, T. Xanthos, N. Iacovidou

**Affiliations:** ^1^Department of Surgery, Section of Pathology, University of Cagliari, Via Ospedale, Sardinia, 09100 Cagliari, Italy; ^2^Department of Surgery, Section of Neonatal Intensive Care Unit, Puericulture Institute and Neonatal Section, University of Cagliari, 09100 Cagliari, Italy; ^3^Department of Electrical and Electronic Engineering (DIEE), University of Cagliari, 09100 Cagliari, Italy; ^4^ELPEN Research-Experimental Centre, Athens, Greece; ^5^National and Kapodistrian University of Athens, Medical School, Athens, Greece

## Abstract

*Objective*. Evaluation of neuronal changes in an animal experimental model of normocapnic hypoxia- reoxygenation.* Materials and Methods*. Fifty male piglets were the study subjects; normocapnic hypoxia was induced in 40 piglets and ten were sham-operated (controls). When bradycardia and/or severe hypotension occurred, reoxygenation was initiated. Animals were allocated in 4 groups according to the oxygen concentration, they were resuscitated with 18%, 21%, 40%, and 100% O_2_. Persisting asystole despite 10 minutes of cardiopulmonary resuscitation and return of spontaneous circulation were the endpoints of the experiment. Surviving animals were euthanized and brain cortex samples were collected, hematoxylin and eosin-stained, and examined for apoptotic bodies observing 10 consecutive high power fields.* Results*. Histological examination of the control group did not show any pathological change. On the contrary, apoptosis of neurons was found in 87.5% of treated animals. When specimens were examined according to the oxygen concentration used for resuscitation, we found marked intergroup variability; a higher percentage of apoptotic neurons was observed in piglets of group 4 (100% oxygen) compared to the others (*P* = 0.001).* Conclusions*. This preliminary data shows that normocapnic hypoxia and reoxygenation in Landrace/Large White piglets resulted in significant histological changes in the brain cortex. The degree of pathological changes in cortical neurons was significantly associated with the oxygen concentration used for reoxygenation, with a higher percentage of apoptotic neurons being observed in piglets reoxygenated with 100% compared to 18% O_2_ and to 21% O_2_.

## 1. Introduction

The pathogenesis of brain damage induced by hypoxia at birth has not yet been fully elucidated. Neuronal damage has been related to the increased expression of proapoptotic genes, leading to an increased potential for apoptotic cell death in the hypoxic newborn brain [1]. In the brain of newborn piglet, the Bcl-2/Bax ratio was lower in the striatum of asphyxiated animals, suggesting that this brain region may be more susceptible to apoptotic injury. On the other hand, no significant differences in the expression of caspase-3 were detected between SHAM-operated, hypoxic, and ischemic groups [2]. Recent studies have attributed a possible role for poly(ADP-ribose) polymerase-1 (PARP-1), whose overactivation could induce neuronal cell death, whereas its inhibition by nicotinamide might protect against neuronal damage induced by perinatal asphyxia [3].

Previous studies suggested that administration of 100% oxygen was associated with a more favourable outcome than 21% oxygen [4]; in hypothermic newborn piglets which underwent cerebral ischemia, reoxygenation with 21% oxygen showed lower mean arterial pressure and a significantly greater degree of hypoperfusion in cerebral cortex, compared with newborn piglets treated with 100% oxygen, suggesting a less favourable outcome in the group receiving room air [4]. The more favourable outcome in animals resuscitated with 100% oxygen was confirmed by another study in a neonatal piglet model where striatum and hippocampus were better protected from apoptosis of brain cells when 100% rather than 21% oxygen was used during resuscitation [5]. However, recent studies suggest that room air administration may protect the brain [6] and other organs better than 100% oxygen [7].

Based on these data, the present study aimed at investigating the effects of resuscitation with different oxygen concentrations on the apoptotic cell death of brain cortex neurons in a previously described model of normocapnic hypoxia-reoxygenation in newborn piglets [8].

## 2. Material and Methods

Fifty male Landrace/Large White piglets, 1–4 days old, weighing 2.3–3.8 kg, were the study subjects, ten of which served as the control group. The animals were transported to the laboratory (Experimental Research Center ELPEN) on the day of the experiment, all from the same breeding unit (N. Validakis, Koropi, Greece).

The experimental protocol was approved by the General Directorate of Veterinary Services (Permit number 404/21-04-09) and has been previously described [8]. All animals, in the control and experimental groups, were initially sedated with intramuscular administration of 10 mg/kg ketamine (Narketan, Vétoquinol UK Ltd.) and 0.5 mg/kg midazolam (Dormicum, Hoffmann-La Roche, Germany). The marginal auricular vein was catheterized with an appropriate size catheter (Jelco R., Smiths Medical, N. Papapostolou SA, Athens, Greece) and induction of anesthesia was performed with administration of 1 mg/kg propofol (Diprivan, AstraZeneca) and 10 *μ*g/kg fentanyl (Fentanyl, Janssen-Cilag). Endotracheal intubation with a size 3.0 or 3.5 mm endotracheal tube (Portex, Smiths Medical, UK) ensued and auscultation and capnography (Datex Engstrom, Type TC 200-22-01, Instrumentarium Corp., Helsinki, Finland) confirmed correct placement of the endotracheal intubation. Infusion of 10 mL/kg/h NaCl 0.9% and 5 mL/kg/h dextrose in water 5% was administered in order to prevent dehydration and hypoglycemia. Noninvasive continuous monitoring included recording of heart rate (HR), electrocardiogram (ECG), saturation of oxygen by pulse oximeter (SpO_2_), and rectal temperature (Matron, BPM 1000, VET, ET Medical Devices Spa). Special attention was paid to maintaining body temperature at 38 ± 1°C, with the aid of a table-heating pad and an overhead-heating lamp. Intravenous boluses of 20 *μ*g/kg fentanyl and 0.2 mg/kg cisatracurium (Nimbex, Abbott) were administered to the animals, which were then mechanically ventilated (Soxil, Soxitronic, Felino, Italy) (tidal volume of 10–15 mL/kg, pressure of 19 cm H_2_O, and respiratory rate of 30–40 breaths/minute, aiming at end-tidal CO_2_ (ETCO_2_) of 35–45 mm Hg). The fraction of inspired oxygen (fiO_2_) was adjusted between 0.21 and 0.25 aiming at SpO_2_ 90–95%. Anesthesia was maintained by infusion of 8–10 mg/kg/h propofol and boluses of 10 *μ*g/kg fentanyl and 0.15 mg/kg cisatracurium.

Subsequently, via a paratracheal incision, the right internal jugular vein and carotid artery were catheterized, with single-lumen catheters (S1UVC5.0, NeoCare; Klein-Baker Medical Co., San Antonio, TX, USA). External transducers (Transpac, Abbott Critical Care Systems, USA) connected to the catheters monitored continuously central venous pressure (CVP) and systolic (SAP), mean (MAP), and diastolic pressure (DAP) of the carotid artery. The incision was sutured and covered with sterile warm gauzes to prevent heat loss. A 30-minute period prior to experimentation was allowed to animals for stabilization.

Hypoxia was induced only in the experimental group by lowering the inspired fiO_2_ to 0.06–0.08, while maintaining the other settings of ventilation. Close monitoring was carried out to detect either bradycardia (HR < 60 beats per minute) or severe hypotension (MAP < 15 mm Hg). The time required for these to occur was recorded. As soon as hemodynamic compromise occurred and arterial blood gases confirmed hypoxemia (pO_2_ 30–50 mm Hg), resuscitation efforts were initiated according to the Newborn Life Support (NLS) algorithm. Animals were allocated in 4 groups to receive different O_2_ concentration for resuscitation: groups 1, 2, 3, and 4 receiving 18%, 21%, 40%, and 100% O_2_, respectively, until HR and MAP returned to 90% of baseline levels. As soon as these hemodynamic parameters returned to baseline values, the piglets were further ventilated (still under anesthesia) for 30 minutes. Persisting asystole despite 10 minutes of cardiopulmonary resuscitation or return of the hemodynamic parameters to baseline values were the endpoints of the experiment. Surviving animals were humanely euthanatized while still under general anesthesia by slow intravenous infusion of 30 mg/kg sodium thiopental (Pentothal, Hospira Enterprises BV, The Netherlands). Necropsy followed for examination of possible injury or abnormality.

Multiple brain frontal cortex samples were collected from both groups of animals (control and experimental),* in toto*, reduced, fixed in 10% formalin, routinely processed, and paraffin-embedded; the initial block was cut into 6-7 blocks of about 2-3 mm wide that were previously deparaffinized with xylene and hydrated. They were then colored with hematoxylin for 2 minutes, washed in running tap water for 20 minutes, and counterstained with eosin from 15 seconds to 2 minutes. Finally slides were dehydrated in 95% absolute alcohol and cleared in xylene.

To minimize errors due to sampling variability, the count of apoptotic cells was based on the observation of five high power fields, randomly selected from all the zones of the collected brain samples.

### 2.1. Statistical Analysis

Statistical analysis was done with Mathworks MATLAB for Mac (version R2009a). The analysis was performed with an ANOVA for repeated measure, using group as between-subject factor and both neuronal layer and field as within-subject factor (Greenhouse-Geisser corrected). Tukey's honest significance post hoc ANOVA test was applied to further evaluate differences in treatment levels. A significance level of alpha <0.05 was used for all tests.

## 3. Results

**Figure 1:**
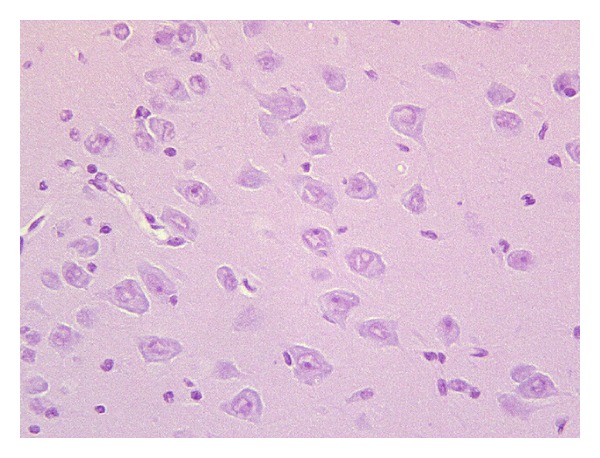
Brain cortex of control animals. Cortical neurons are characterized by large nuclei with prominent nucleoli (400x).

At histology, the brain cortex of the control piglets did not show any pathological changes. Cortical neurons, organized into 4 to 5 layers, were characterized by a large cytoplasm and a voluminous nucleus with open chromatin (Figure 1). No apoptotic cells were detected in all brain cortex samples analysed.

**Figure 2:**
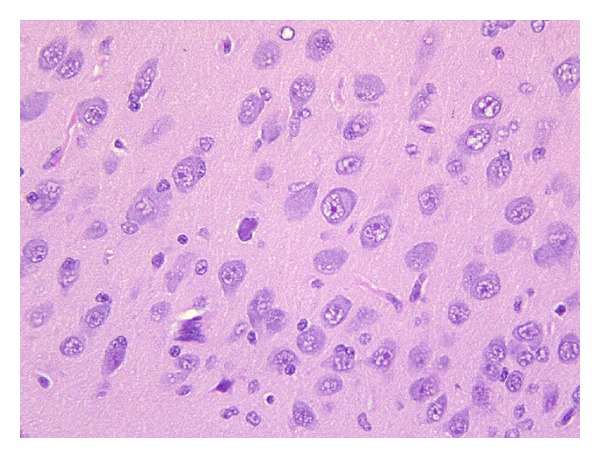
Brain cortex of group 1 piglets. Among the vast majority of normal neurons, scattered cells (arrows) appear hyperchromatic and show shrinkage of the cell body, all representing morphological evidence of apoptotic cell death (400x).

**Figure 3:**
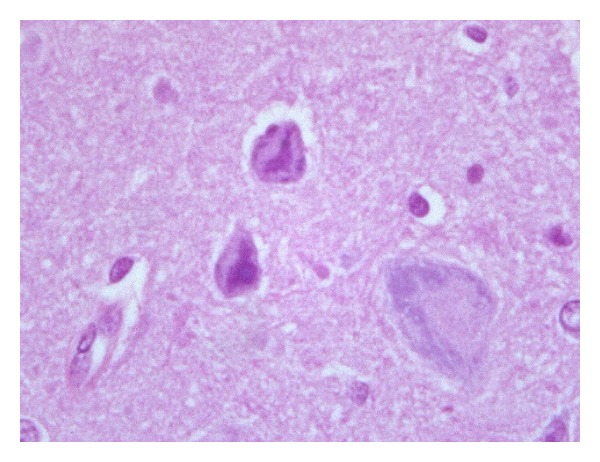
Brain cortex of group 1 piglets. At high power, neurons undergoing apoptosis show relevant nuclear changes with chromatin condensation and nuclear pyknosis (800x).

**Figure 4:**
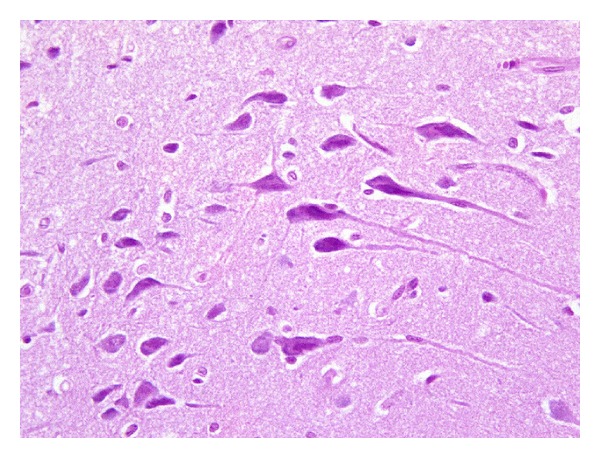
Brain cortex of group 4 piglets. Cortical neurons of animals resuscitated with 100% oxygen show diffuse apoptosis, in the vast majority of neuronal cells (400x).

Contrarily, hypoxic piglets exhibited significantly different histological picture of their brain cortices compared to that of control animals. In the vast majority of cases, the principal pathological change was apoptosis of neurons. Neurons undergoing apoptotic cell death were characterized by condensation of the cytoplasm, which resulted in a hypereosinophilic cell. Hypereosinophilia was followed by cell shrinkage and chromatin condensation, resulting in hyperchromatic nuclei (Figure 2). Apoptotic neurons were, in the majority of cases, scattered, intermingled among adjacent normal neurons (Figure 3). In some cases, apoptosis appeared more diffuse, interestingly in a large number of cortical neurons (Figure 4). Apoptotic cells were observed both in the superficial and in the deep cortical layers. Cerebral oedema was present in a minority of cases. No significant vascular changes were detected.

The distribution of apoptotic bodies, in each brain cortex sample analysed, was uneven, with some cortical areas containing a large number of neurons undergoing apoptotic cell death, adjacent to other areas characterized by the absence of apoptotic neurons.

**Figure 5:**
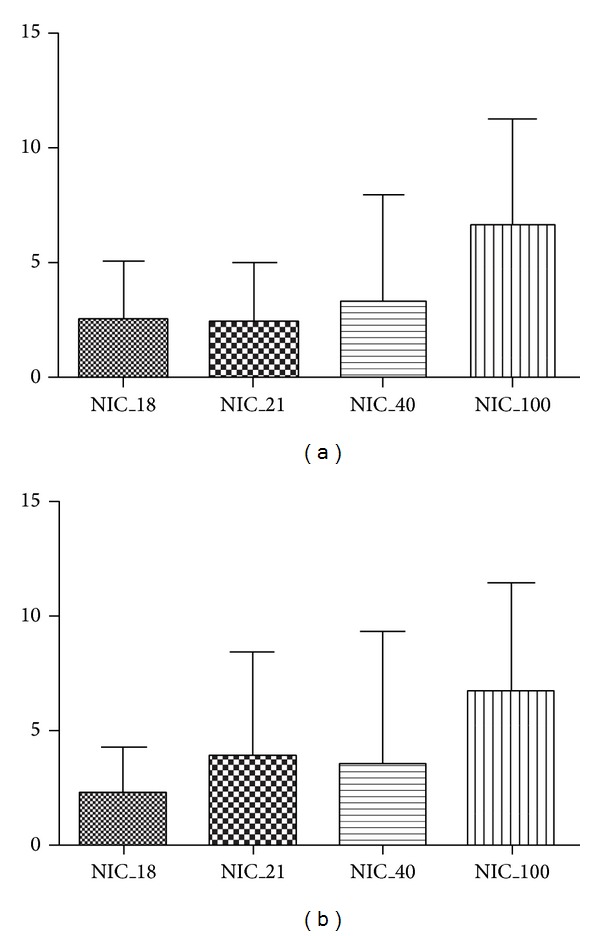
Statistical analysis using ANOVA for repeated measures. Apoptosis of cortical neurons is significantly increased in group 4 animals (NIC_100) as compared with group 1 (NIC_18) and group 2 (NIC_21) piglets.

When specimens were examined according to the oxygen concentration they were resuscitated with, marked differences were detected among the groups as to the extent of apoptosis of brain cells. In particular, a higher percentage (59.2%) of apoptotic neurons were found in piglets of group 4 (100% oxygen) compared to those of the other groups (Figure 5). A marked interindividual variability among animals of the same group, in all 4 groups, was also detected.

Statistical analysis using ANOVA for repeated measures showed significant differences between group 1 and group 4 (*P* = 0.01) and between group 2 and group 4 (*P* = 0.047). In particular higher levels of apoptosis were observed in group 4.

## 4. Discussion

Despite advances in neonatal intensive care over the last decades, hypoxia at birth represents one of the most important causes of brain damage in term [9, 10] and preterm neonates [11], and a major medico-legal problem even in developed countries. Increased perinatal morbidity and mortality is associated with the condition and long-term neurological deficits have been reported in survivors [12]. It has been estimated that 25 per 1,000 live births at term and 73 per 1,000 live births in the preterm experience some degree of perinatal hypoxia [13].

The mechanism by which perinatal asphyxia causes neuronal cell damage, and damage in other organ cells, has not yet been fully elucidated. Recently, several hypotheses have been proposed. The role of hypoxia in determining brain damage can accurately be investigated in animal models, with the piglet model being one of the most widely used ones [14]. In these studies, the vulnerability of brain cells to hypoxia/reoxygenation was mainly related to the following two factors: (i) the decreased availability of oxygen due to inadequate microcirculatory delivery; (ii) the endothelial dysfunction in cortical vessels leading to increased vascular permeability, leakage from the vascular bed towards the surrounding tissue, interstitial oedema, and cerebral congestion. Recently, metabolomics in plasma of piglets submitted to hypoxia/resuscitation allowed the identification of a third factor, which is the oxidative stress caused by resuscitation with 100% oxygen which seems to delay cellular recovery after asphyxia [15].

The present study suggests that hypoxia, at histology, induces apoptotic cell death in the neurons of the brain cortex of newborn piglets. Our data are in line with previous studies demonstrating an increased expression of proapoptotic proteins [6] and changes in the Bcl-2/Bax [7] ratio in the cerebral cortex of newborn pigs following hypoxia.

Another interesting finding of the present study is the high degree of variability in the extent of apoptotic neuronal cell death among the 4 groups of piglets. The number of neurons undergoing apoptosis was higher in group 4. Statistical analysis revealed a significant difference in the amount of apoptotic cortical neurons observed in specimens between group 1 and group 4 piglets (Figure 5(a)). The difference in the amount of apoptotic bodies was statistically significant between animals of group 2, compared to those of group 4 (Figure 5(b)).

This finding deserves some considerations. Conflicting results have been reported in recent years regarding the safety of oxygen during resuscitation at concentrations higher than 21%. On one hand, it has been claimed that reoxygenation with room air could be associated with a significantly greater degree of hypoperfusion in cerebral cortex, compared with newborn piglets treated with 100% oxygen, suggesting a less favourable outcome in this group [9]. The possible more favourable outcome in animals treated with 100% oxygen was observed in another study in piglets: striatum and hippocampus were shown to be better protected against apoptosis of brain cells when resuscitation was conducted with 100% rather than 21% oxygen [10]. On the other hand, recent studies based on metabolomics reported that resuscitation with 100% oxygen caused a delay in cellular recovery after hypoxia [15]. Our data confirms, at histological level, metabolomics studies showing that oxygen during resuscitation of asphyxiated newborns in clinical practice should be used judiciously, according to the significantly higher degree in neuronal cell death observed in piglets resuscitated with 100% as compared to those resuscitated with air room. Moreover, our data support the hypothesis that brain damage following hypoxia might be negatively influenced by free radicals and that the use of oxygen in high concentrations might be more neurotoxic than room air in hypoxic newborn piglets [16, 17].

Finally, the marked interindividual variability as to the extent of apoptotic cell death, previously reported by our group in kidneys [18] and hearts [19], is observed in the neurons of the brain cortex as well. This interindividual variability was observed within all 4 groups, suggesting a major role for the individual susceptibility of each animal to hypoxia/reoxygenation-related brain damage. This datum suggests, in clinical practice, the need for a tailored approach to each newborn affected by perinatal postasphyctic syndrome, with the aim of inhibiting both the pathological effects caused by hypoxia and neural cell death caused by oxygen stress during resuscitation. In particular, the effectiveness of the antagonists of free radicals in inhibiting oxidative stress of cortical neurons should be considered, in accordance with the positive effects reported by the use of the thiophosphate WR.2721 in critically ill patients affected by hypoxic renal dysfunction [20].

Further studies are needed to confirm our data, mainly based on morphology, at immunohistochemical level by the study of apoptotic marker proteins and at molecular level using immunoblotting analyses of tissue mRNA/protein expression and of oxidative stress markers. The long-term consequences of the acute brain neuronal damage in the hypoxia/reoxygenation piglet model hereby described should be also investigated in order to better understand the consequences of the posthypoxic apoptotic changes evidenced in this study on brain functional activity in childhood and adulthood.

## References

[1] Delivoria-Papadopoulos M, Ashraf QM, Mishra OP (2007). Differential expression of apoptotic proteins following hypoxia-induced CREB phosphorylation in the cerebral cortex of newborn piglets. *Neurochemical Research*.

[2] Pirzadeh A, Mammen A, Kubin J (2011). Early regional response of apoptotic activity in newborn piglet brain following hypoxia and ischemia. *Neurochemical Research*.

[3] Allende-Castro C, Espina-Marchant P, Bustamante D (2012). Further studies on the hypothesis of PARP-1 inhibition as a strategy for lessening the long-term effects produced by perinatal asphyxia: effects of nicotinamide and theophylline on PARP-1 activity in brain and peripheral tissue—nicotinamide and theophylline on PARP-1 activity. *Neurotoxicity Research*.

[4] Solas AB, Kutzsche S, Vinje M, Saugstad OD (2001). Cerebral hypoxemia-ischemia and reoxygenation with 21% or 100% oxygen in newborn piglets: effects on extracellular levels of excitatory amino acids and microcirculation. *Pediatric Critical Care Medicine*.

[5] Mendoza-Paredes A, Liu H, Schears G (2008). Resuscitation with 100%, compared with 21%, oxygen following brief, repeated periods of apnea can protect vulnerable neonatal brain regions from apoptotic injury. *Resuscitation*.

[6] Saugstad OD (2010). Resuscitation of newborn infants: from oxygen to room air. *The Lancet*.

[7] Vento M, Sastre J, Asensi MA, Viña J (2005). Room-air resuscitation causes less damage to heart and kidney than 100% oxygen. *American Journal of Respiratory and Critical Care Medicine*.

[8] Aroni F, Xanthos T, Varsami M (2012). An experimental model of neonatal normocapnic hypoxia and resuscitation in Landrace/Large white piglets. *Journal of Maternal-Fetal and Neonatal Medicine*.

[9] Rivkin MJ (1997). Hypoxic-ischemic brain injury in the term newborn: neuropathology, clinical aspects, and neuroimaging. *Clinics in Perinatology*.

[10] Al-Macki N, Miller SP, Hall N, Shevell M (2009). The spectrum of abnormal neurologic outcomes subsequent to term intrapartum asphyxia. *Pediatric Neurology*.

[11] Schneider H (1995). Birth asphyxia as a cause of childhood brain damage?. *Archives of Gynecology and Obstetrics*.

[12] Lawn J, Shibuya K, Stein C (2005). No cry at birth: global estimates of intrapartum stillbirths and intrapartum-related neonatal deaths. *Bulletin of the World Health Organization*.

[13] Low JA (2004). Determining the contribution of asphyxia to brain damage in the neonate. *Journal of Obstetrics and Gynaecology Research*.

[14] Feet BA, Yu X-Q, Rootwelt T, Öyasaeter S, Saugstad OD (1997). Effects of hypoxemia and reoxygenation with 21% or 100% oxygen in newborn piglets: extracellular hypoxanthine in cerebral cortex and femoral muscle. *Critical Care Medicine*.

[15] Solberg R, Enot D, Deigner H-P (2010). Metabolomic analyses of plasma reveals new insights into asphyxia and resuscitation in pigs. *PLoS ONE*.

[16] Atzori L, Noto A, Barberini L, Iacovidou N, Marinelli V, Fanos V, Fanos V, Chevalier RL, Faa G, Cataldi L (2011). Metabolomics in perinatal renal asphyxia. *Developmental Nephrology: Form Embryology To Metabolomics*.

[17] Biban P, Filipovic-Grcic B, Biarent D, Manzoni P (2011). New cardiopulmonary resuscitation guidelines 2010: managing the newly born in delivery room. *Early Human Development*.

[18] Gerosa C, Iacovidou N, Argyri I Histopathology of renal asphyxia in newborn piglets: individual susceptility to tubular changes.

[19] Faa A, Iacovidou N, Xanthos T (2012). Hypoxia reoxygenation-induced myocardial lesions in newborn piglets are related to interindividual varaiability and not to oxygen concentration. *Clinics*.

[20] Pedrotti A, Bonjour J-P, Guignard JP (1992). Protection from hypoxemia-induced renal dysfunction by the thiophosphate WR-2721. *Kidney International*.

